# Non-invasive Biofouling Monitoring to Assess Drinking Water Distribution System Performance

**DOI:** 10.3389/fmicb.2021.730344

**Published:** 2021-10-28

**Authors:** Frances C. Pick, Katherine E. Fish, Stewart Husband, Joby B. Boxall

**Affiliations:** Department of Civil and Structural Engineering, Sheffield Water Centre, The University of Sheffield, Sheffield, United Kingdom

**Keywords:** biofouling, biofilms, water quality, drinking water distribution systems, flow cytometry

## Abstract

Biofilms are endemic in drinking water distribution systems (DWDS), forming on all water and infrastructure interfaces. They can pose risks to water quality and hence consumers. Our understanding of these biofilms is limited, in a large part due to difficulties in sampling them without unacceptable disruption. A novel, non-destructive and non-disruptive biofilm monitoring device (BMD), which includes use of flow cytometry analysis, was developed to assess biofouling rates. Laboratory based experiments established optimal configurations and verified reliable cell enumeration. Deployment at three operational field sites validated assessment of different biofouling rates. These differences in fouling rates were not obvious from bulk water sampling and analysis, but did have a strong correlation with long-term performance data of the associated networks. The device offers the potential to assess DWDS performance in a few months, compared to the number of years required to infer findings from historical customer contact data. Such information is vital to improve the management of our vast, complex and uncertain drinking water supply systems; for example rapidly quantifying the benefits of improvements in water treatment works or changes to maintenance of the network.

## Introduction

Biofilms are endemic within drinking water distribution systems (DWDS) and can impact water quality, yet current water quality sampling and monitoring is typically restricted to the easily accessible bulk water, which is not representative of the quantity or composition of biofilms at the pipe wall. As the dominant form of microbial loading within DWDS, biofilms drive pipe-wall and water quality interactions including hosting the accumulation of particulate material, which is a source of discolouration and associated high metal concentrations if mobilised (Ginige et al., 2011; Husband and Boxall, 2011; Benson et al., 2012; Fish et al., 2016).

Discolouration events are a leading example of distributed water quality failure that occur worldwide. They impact consumer confidence and as a major cause of customer contacts, they are used as a service indicator in the United Kingdom, with performance linked to financial rewards or penalties (Vreeburg and Boxall, 2007; OFWAT, 2021). In 2019, the United Kingdom drinking water industry received an average of 1.25 customer contacts per 1,000 population for appearance and taste/odour, with 55% of all contacts reporting a concern with water quality being related to discoloured water (Drinking Water Inspectorate [DWI], 2019). Discolouration has been described as a three-step process consisting of low-level material supply, the accumulation of this material on pipe surfaces and then its rapid mobilisation into the bulk water (Boxall et al., 2001). This behaviour has been verified worldwide in the empirical PODDS (Prediction of Discolouration in Distribution Systems) model (Husband and Boxall, 2011) and is analogous to biofilm behaviour (Husband et al., 2016) with biofilms central in governing the rate and cohesive strength of the material that accumulates (Abe et al., 2012; Mathieu et al., 2014; Aggarwal et al., 2015; Gerbersdorf et al., 2020).

Biofilms are a complex arrangement of microorganisms adhered to a surface via a matrix of microbially-derived extracellular polymeric substances (EPS), within which trace inorganics such as iron are known to accumulate (Fish et al., 2020). Biofilms present in DWDS can be the result of years of growth and influenced by historic operational practices, existing infrastructure and the ecology of complex microbial communities. The growth rate of biofilms is known to be affected by physical and chemical parameters such as water source (Douterelo et al., 2016), organic (Pick et al., 2021), and inorganic loading (Chu et al., 2005), chlorine concentrations (Fish et al., 2020) and temperature (Hallam et al., 2001). Following a 12-month biofouling period, Pick et al. (2021) reported a positive relationship between turbidity, inorganic concentrations and cell counts being released into the bulk water due to increasing shear stress at the pipe wall mobilising biofilm. It is proposed that a robust, repeatable method for quantification of biofouling rates could be used as an indicator of the discolouration, and other water quality, potential within DWDS.

There are currently no recognised methods available to monitor DWDS biofilms *in situ*, due in a large part to the buried and inaccessible nature of DWDS pipes. As a result, current microbial monitoring is usually restricted to planktonic microorganisms via analysis of easily accessible bulk-water samples, or biofilms within bench top scale systems. Some previous studies have analysed adenosine triphosphate (ATP) to determine the active biomass, and hence biofouling rate, of biofilms grown on beads in glass cylinders connected to DWDS (Van der Kooij and Veenendaal, 1993; Van der Kooij et al., 2003). Other studies have reported difficulties with the application of ATP to drinking water environments, in particular when a chlorine residual is present (Pick et al., 2019). Flow cytometry has been successfully used to enumerate planktonic cells within water samples and discriminate between total and intact (more likely viable) cells (Hammes et al., 2008; Gillespie et al., 2014; Fish et al., 2020; Pick et al., 2021). Techniques using flow cytometry have also been applied to biofilms to provide a quick and reproducible method for DWDS biofilm cell enumeration (Fish et al., 2020), but have relied on network invasive strategies using pipe coupons to obtain biofilm samples.

Bench-top systems such as flow cells, reactors or small-scale pilot pipe systems can provide access for biofilm sampling but are often inaccurate representations of the pipeline environment, especially with respect to DWDS hydraulic conditions. Thus, there are difficulties in translating biofouling rates (known to be impacted by the DWDS pipe environment) from studies at these scales to the real world. A number of devices, such as Pennine Water Group (PWG) coupons (Deines et al., 2010), have the potential to be installed within drinking water pipes or bypasses. These devices have been trialled in DWDS; however, there are obstacles for wide scale implementation due to difficulties from the invasive fitting and collection processes, and the need for permanent chambers to provide long term access to pipe lengths. Consequently, there is a need for non-invasive, repeatable and simple to install techniques to monitor biofouling in DWDS in such a way that useful data can be easily and readily collected for use by water suppliers.

This research aimed to develop a robust method to study and quantify biofouling rates within operational DWDS. Specific objectives were to develop, optimise and verify the design, configuration and installation specifications of a non-destructive and non-disruptive, easily deployable biofilm monitoring device (BMD) to assess biofouling rates in full-scale laboratory conditions. Once established the research would set out to validate the operational use of the BMD in the field, via installation in networks with different water qualities. The device aimed to differentiate biofouling rates based on bulk water quality at a given location, rather than replicating conditions within specific pipe. The value of the BMD could then be assessed by comparing results obtained from the BMD to insights provided by historic water quality and customer contact data.

## Methodology and Methods

### Biofilm Monitoring Device Design

**FIGURE 1:**
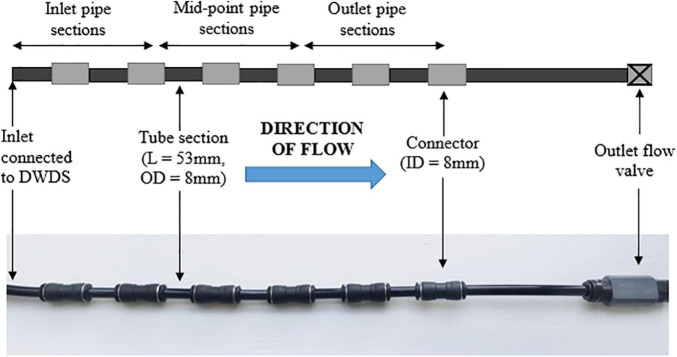
Illustration of the biofilm monitoring device (BMD), including tube sections, connectors and a flow valve on the BMD outlet. L, length; OD, outside diameter; ID, inner diameter. BMD diagram is an illustration, and does not show the full number of tube sections used in an installation.

The main advantage of the BMD, in comparison to other *in situ* monitoring devices, is that the device can easily be connected to a tapping point within an operational DWDS, to provide non-destructive and non-disruptive sampling of biofilms. Central to an effective BMD are accessible and representative surfaces for biofilm development. This was achieved by connecting a series of short and identical pipe lengths to an incoming water source. The BMD is comprised of a series of 53 mm long tube sections (8 mm external diameter, Festo Air Hose Black Polyurethane PUN Series, RS Components United Kingdom, number not fixed) that are separated by spacer tubes (8 mm internal diameter Festo QS Pneumatic Straight Tube-to-Tube Adapter, RS Components United Kingdom) (Figure 1). The material of the BMD was designed to be consistent between sites to generate comparable results. A flow valve is attached to the outlet (Akro Valves Ltd., United Kingdom) to maintain a consistent water flow rate and positive pressure within the device. Each BMD is supplied with drinking water directly from the DWDS tapping point. Prior to installation all tube sections and connectors were washed and sterilised via sonication with a 2% (w/v) sodium dodecyl sulfate (SDS) solution (Pick et al., 2021).

### Optimum Biofilm Monitoring Device Configuration: Laboratory Trials

It is vital that conditions within the BMD are sufficiently representative of the environment of interest and that these conditions are consistent over the length of the device (inlet, mid-point, and outlet). Conditions within the BMD are dominated by physical effects such as turbulence regime for nutrient flux and gravitational effects. Laboratory based experiments at the University of Sheffield were used to investigate and optimise these. The BMDs were supplied with water from the local DWDS (surface water source) distributed via a cast iron trunk main directly into the building that houses the testing facility (i.e., no local distribution networks and minimal dedicated premises pipes).

Trials were conducted to determine the optimum configuration of the BMD, including investigating BMD length (number of removable sections), flow rate and orientation. During analysis pipe sections were collected simultaneously from the inlet, middle and outlet of the BMD to investigate longitudinal effects, with consistency of data between removed sections a prime indicator. Three BMDs were installed horizontally and three installed vertically. For each orientation, each BMD was assigned a specific flow rate based on Reynolds number (representing turbulent, transitional, and laminar regimes) to investigate the effect of different hydraulic conditions on biofilm development. The flow rates investigated were 0.5, 1, and 2 L/min (assuming k_*s*_ of 0.03, Re ≈ 1,500, 3,000, and 6,000, respectively) with each flow rate being achieved by a fixed rate flow valve on the BMD outlet. A ks value is required to represent the roughness of the internal surface of the pipe. The flow rates were chosen to enable a comparison between which flow rate provided most consistent measurements along the length of the BMD. The flow rates, and conditions within the BMD were not designed to replicate a specific pipe within a DWDS, rather to assess the biofouling rate as a function of the bulk water quality in a given part of a network. The BMDs were sampled (in triplicate) at 2 weeks, 3 and 6 months to assess the cell counts (total cell count: TCC and intact cell count: ICC) of newly formed, developing and mature biofilm.

### Development of Biofouling Curves: Drinking Water Distribution Systems Installation

#### Site Selection

In order to verify the capability of the BMD to establish a biofouling rate, the BMD was initially installed at The University of Sheffield drinking water laboratory and biofouling was monitored for 12 weeks (Table 1), referred to as Site 1. Subsequently, BMDs were installed at three different operational networks (Site 2, Site 3, and Site 4) to test application by quantifying biofouling rate. All field sites were selected by project partners South Staffordshire Water, as sites of operational interest with contrasting water qualities and suitable sample points within buildings or kiosks to mount the BMDs. Installations across Sites 2–4 were conducted simultaneously with an August start date. At Site 3 a BMD was also installed in November to investigate the effect of water temperature (seasonal variation) on biofouling rates. Site details are given in Table 1.

**TABLE 1:** Summary of the key features of the sites used for investigation into the impact of water quality on the biofouling rate.

**Site name**	**Location**	**Water source**	**Disinfectant type**	**Water age (hours)**	**Distance from WTW (km)**	**Installation**
1	UoS building	Surface water	Chlorine	29.5–67.0 (blend)	10, twin 18” CI trunk main	Laboratory verification
2	SSW building	Ground-water/mix		8.5	6, 18” CI trunk main	August field installation
3	SSW sampling kiosk	Surface water		2.5	5.4, 19.7” DI trunk main	August and November field installation
4				16.0	27, 15.7” DI trunk main	August field installation

*UoS, University of Sheffield; SSW, South Staffordshire Water; WTW, water treatment works; CI, cast iron; DI, ductile iron.*

#### Biofilm Monitoring Device—Biofilm Sampling

Biofilm samples were collected from the BMDs for 12 weeks to generate a biofouling curve with weekly sampling for 4 weeks, followed by sampling every 2 weeks for the remaining 8 weeks. Each BMD consisted of 24 tube sections, with each tube section divided by connectors. During aseptic collection of individual tube sections for sampling (*n* = 3), the flow to the BMD was temporarily switched off (<1 min), before the remaining tube sections were reconnected and flow resumed. One end of the removed tube section was sealed with parafilm and phosphate-buffered saline (PBS) was pipetted into the tube section to ensure the biofilm remained hydrated. The open end of the tube section was then sealed with more parafilm. The BMD were kept cool during transport back to the laboratory for analysis in less than 6 h. Biofilms suspensions were created from tube sections in the laboratory using the brushing technique described in Fish et al. (2020), adapted to use nylon cylinder brushes (LESSMANN, Germany) to remove biofilm from the interior surface of the BMDs into a 30 mL volume of PBS. The biofilm suspensions were then ready for downstream analysis, which was undertaken within 12 h of collection.

#### Bulk Water Sampling

To investigate the impact of incoming bulk water quality on the biofouling rate, bulk water samples were collected whenever biofilm sampling was undertaken. Bulk water parameters measured included TCC, ICC, total chlorine, free chlorine, turbidity, pH, temperature, and ORP (Table 2). All bulk water samples were collected in triplicates of 500 mL and measured *in-situ*, excluding TCC and ICC for which triplicates of 15 mL were collected for analysis in the laboratory. Bulk water samples for planktonic TCC and ICC water were dechlorinated prior to analysis—a 4.5 μL volume of a 1% (w/v) sodium ascorbate solution was added to each 15 mL sample (using a 1% sodium ascorbate solution) (Fish et al., 2020 and Preciado et al., 2021). Air temperature was recorded every 30 s using a LASCAR Electronics USB temperature data logger installed within the sampling kiosks/building at Site 2, Site 3, and Site 4.

**TABLE 2:** Methods used to measure discrete bulk water parameters (*n* = 3) collected during laboratory and/or fieldwork.

**Water quality parameter**	**Instrument/Analysis method**	**Range**	**Resolution**	**Accuracy**
TCC and ICC	C6 flow cytometer (BD Accuri, United Kingdom).	10^2^–10^7^ cells/mL	10,000 events/s and a sample concentration over 5 × 10^6^ cells/mL	−
Total and free chlorine	Palintest chlorosense	0–10 mg/L free chlorine 0–100 mg/L total chlorine	0.01 up to 5.0 mg/L	Free chlorine: 5% CV @ 1.00 mg/L. Total chlorine: 5% CV @ 10 mg/L
Turbidity	Hach 2100Q portable Turbidimeter	0–1,000 NTU	0.01 NTU	±1%
pH temperature ORP	Hanna HI991003 portable multi-probe	−2.00–16.00 pH −5–105°C ±1,999 mV	0.01 pH 0.1°C 1 mV	±0.02 pH ±0.5°C ±2 mV

### Cell Enumeration Using Flow Cytometry

#### Planktonic Total Cell Count and Intact Cell Count

Planktonic TCC and ICC (cells/mL) were measured using the flow cytometry method detailed in Hammes et al., 2008. In summary, 500 μL dechlorinated water samples were stained with 5 μL SYBR Green (Life Sciences, California, United States) for TCC. 500 μL dechlorinated water samples were stained with 6 μL SYBR Green/Propidium Iodine mixture (Life Sciences, California, United States) (with a final concentration of 1x SYBR Green and 3 μM PI) for ICC. Although there are caveats of using Propidium Iodine, which have been highlighted elsewhere (Berney et al., 2007), the protocol in this study is a standard protocol and is well published (Hammes et al., 2008; Gatza et al., 2013; Prest et al., 2014; Fish et al., 2020). Following staining, samples were vortexed for 5 s before being incubated at 36°C for 10 min. Flow cytometry analysis was performed using a run limit volume of 50 μL and a medium fluidics speed. Raw cell counts (count/μL) were converted to TCC/ICC per mL, by first multiplying cell counts by the sample volume (50 μL), before multiplying by 1,000 to convert TCC/ICC per μL to TCC/ICC per mL. Samples were analysed using BD Accuri C6 Flow Cytometer with autosampler (BD Accuri, United Kingdom), with a fixed gate plot as described in Fish et al. (2020). The flow cytometry template used to analyse planktonic and biofilm samples included a singlet doublet analysis which enabled a quantitative assessment of sample homogenisation (Fish et al., 2020). In all samples, ≥98% of the data were singlets demonstrating that the samples were well homogenised. All appropriate negative controls were performed, including negative controls for stains, and calibration beads were run daily.

#### Biofilm Total Cell Count and Intact Cell Count

A 500 μL volume of each biofilm suspension was stained and analysed in accordance with the flow cytometry protocol described in section “Planktonic TCC and ICC.” To convert the cell counts into cell concentrations (ICC/7mm^2^ or TCC/mm^2^), Equation 1 was used:


(1) ICC or TCC=((CountVolumeanalysed)×Total volume of sample)Surface Area

Where the count is the total or intact cell count, volume analysed is the volume of sample that was processed in the flow cytometer (50 μL), the total volume of samples in this case was 30 mL (30,000 μL) and the surface area is the area from which the biofilm was removed (915.78 mm). All raw biofilm data was converted into ICC/mm^2^ or TCC/mm^2^. Preliminary tests of technical replication showed no difference, so only biological replication samples were undertaken (*n* = 3).

### Historical Water Quality Data

Historical water quality and customer contact data were supplied by South Staffordshire Water to allow investigation of the biofouling rates results by correlating with established performance measures. Distribution Management Areas (DMAs) directly associated with the BMD sites were identified by South Staffordshire water, based on GIS data and hydraulic modelling. DMAs providing an approximately similar total population served for each BMD site were selected, but having different total pipe lengths (Site 2: 4,342 population, 45 km pipe length; Site 3: 5,021 population, 24 km pipe length; Site 4: 6,226 population, 63 km pipe length). Regulatory water quality data for the last 6 years was analysed, including iron, manganese, turbidity, plate counts, water temperature, free chlorine, and total chlorine data from these DMAs. Customer contacts numbers and dates for these DMAs for the last 6 years were also collated.

### Data Analysis

The mean, median, range, and standard deviation were calculated for each of the bulk-water quality parameters listed in Table 2. The normality of biofilm and water quality data was analysed using the Shapiro-Wilks test and parametric (ANOVA and Tukey) or non-parametric tests (Kruskal Wallis and two-sample Wilcoxon) were applied, as appropriate, to identify any differences in water quality parameters or biofilm cell concentrations between experiments, sites, or sample points. All statistical analysis and graphical plots were generated in R v3.5.2 (R Foundation for Statistical Computing Platform, 2018) with a significance level of *p* < 0.05.

## Results

### Optimum Biofilm Monitoring Device Configuration

**FIGURE 2:**
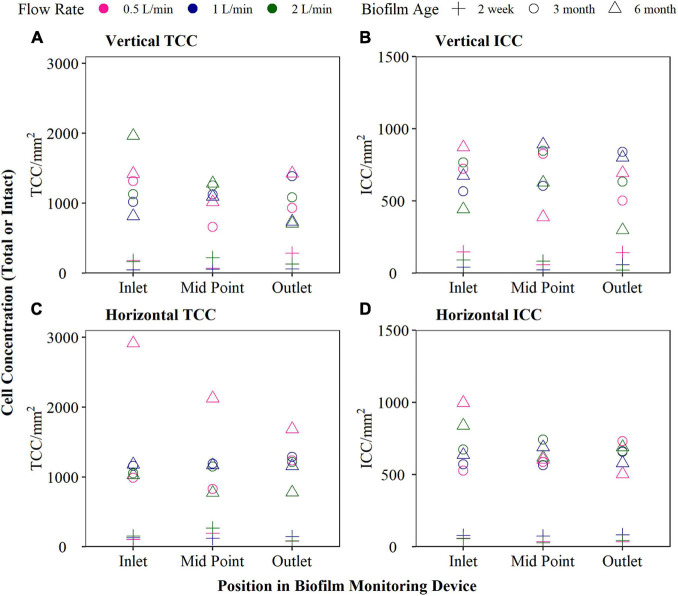
Biofilm cell counts at 2 week, 3 and 6 month biofilm accumulated within biofilm monitoring devices (BMD) installed horizontally or vertically and operated at a flow rate of either 0.5, 1, or 2 L/min. Total cell count (TCC) and intact cell count (ICC) results are plotted (sampled from the inlet, mid-point and outlet of the BMD).

Newly formed (2 weeks), developing (12 weeks), and mature biofilms (6 months) were analysed under three different flow rates (0.5, 1, and 2 L/min), and both horizontal and vertical orientations (Figure 2). The horizontal BMD, supplied by drinking water with a flow rate of 1 L/min, exhibited the least variation in both TCC and ICC along the length of the BMD (as assessed using the variance). Greater variation was seen for all flow rates in the vertical orientation, compared to the horizontal orientation. Additionally, longitudinal variation was detected at 0.5 L/min, particularly in terms of TCC, for both orientations. Therefore, the optimal configuration identified and hence used for all future laboratory and fieldwork was horizontal with a flow rate of 1 L/min. Although the flow rate of 2 L/min also demonstrated little longitudinal variation, the water savings from the lower flow rate was considered preferable.

### Verification of Biofilm Monitoring Device at Site 1

**FIGURE 3:**
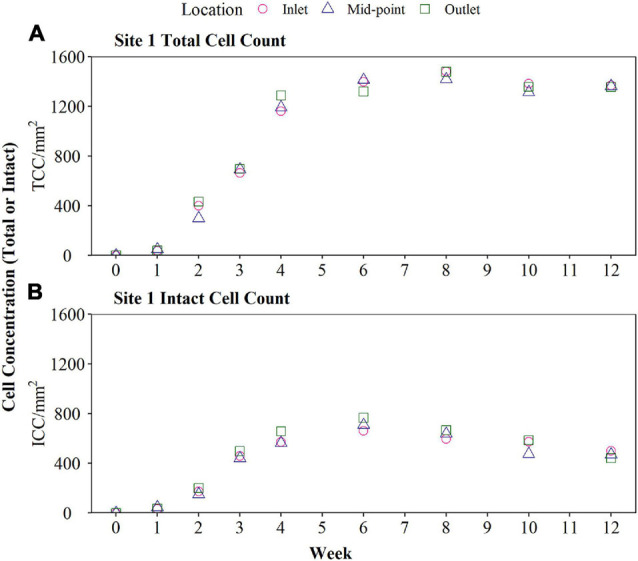
Verification of biofilm monitoring device (BMD) installed at Site 1 for 12 weeks in October. Biofilm cell count data, including total (TCC) and intact cell count (ICC), obtained from BMD are presented (raw data plotted including sampling from the inlet, mid-point, and outlet of the BMD). The BMD was installed horizontally and operated with a flow rate of 1 L/min.

The biofilm cell concentrations (TCC and ICC) for Site 1 are shown in Figure 3, demonstrating that the BMD successfully generated a fully quantifiable biofouling curve after 12 weeks of sampling. The aim of this research was not to develop a filly grown biofilm representative of a particular DWDS, but instead produce comparable biofilm growth curves or biofouling rates that was indicative of the incoming water quality, and DWDS performance. There was no impact of sample location on biofilm TCC (*p* = 0.98) or ICC (*p* = 0.86), as demonstrated by comparing the three tube sections from inlet, middle, and outlet of the BMD, supporting results from the optimisation tests. TCC rapidly increased to 1,286 cells/mm^2^ by Week 4 and continued to increase at a slower rate until reaching a peak TCC count (1,477 cells/mm^2^) at Week 8. TCC remained relatively constant from Week 8 to Week 12 suggesting that the TCC had reached a plateau. Similarly, ICC quickly increased to 658 cells/mm^2^ by Week 4, before reaching the peak ICC (767 cells/mm^2^) at Week 6. Unlike TCC, the biofilm ICC declined from Weeks 6 to 12, indicating a reduction of intact cells in the biofilm. Bulk water quality was analysed during biofilm sampling, resulting data is shown in Table 3.

**TABLE 3:** Bulk water quality supplying the biofilm monitoring devices at Sites 1, 2, 3, and 4.

	**Site 1**		**Site 2**		**Site 3**		**Site 4**	
**Parameter**	**Median**	**Range**	**Median**	**Range**	**Median**	**Range**	**Median**	**Range**
TCC (cells/mL)	1,894	1,478–2,634	19,660	1,340–317,777	9,640	1,200–1,701,299	17,430	404–361,697
ICC (cells/mL)	250	210–405	3,665	800–291,782	2,590	460–230,591	12,250	3,528–75,520
Total chlorine (mg/L)	1.04	0.82–1.30	0.43	0.28–1.0	0.74	0.46–1.33	0.41	0.32–0.80
Free chlorine (mg/L)	0.67	0.62–0.70	0.21	0.02–0.41	0.42	0.27–0.62	0.25	0.03–0.51
Temperature (°C)	9.5	9.3–11.0	13.7	12.2–15.0	14.4	11.2–17.0	15.2	11.6–17.6
pH	6.84	6.45–7.24	6.78	6.47	6.42	6.26–6.72	6.71	6.20–7.29
ORP (millivolts)	595	594–595	505	445–563	502	440–519	481	425–539
Turbidity (NTU)	*	*	0.16	0.01–1.50	0.15	0.01–1.32	0.3	0.01–1.20

*Median and range of bulk water parameters collected in triplicate are listed. TCC, total cell count; ICC, intact cell count. Site 1 water quality was sampled for 12 weeks from November to January and Sites 2, 3, and 4 were sampled for 12 weeks from August to November. ^∗^Turbidity not sampled at Site 1 due to equipment failure.*

### Validation of Biofilm Monitoring Device at Operational Field Sites

**FIGURE 4:**
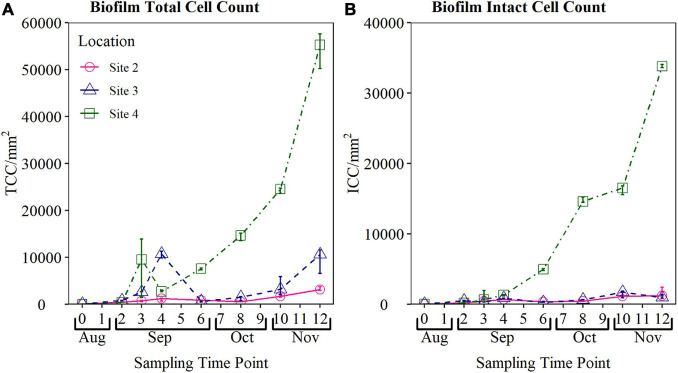
Validation biofouling curves obtained from biofilm monitoring device (BMD) installed at operational field sites. Total cell count (TCC) and intact cell count (ICC) of the biofilm sampled from each BMD are plotted. Biofilm samples were collected weekly for the first 4 weeks, and every 2 weeks from Weeks 4 to 12 (n-3 in all cases). TCC, total cell count; ICC, intact cell count. Note the difference in the *y*-axis scale between **(A,B)**.

The biofouling curves obtained from Site 2, Site 3, and Site 4 from August to November are presented in Figure 4. Results show that statistically different (*p* < 0.05 for both TCC and ICC) biofouling rates were observed at each site, evidence that the device was able to quantify different biofouling at these sites.

The highest peak cell counts were observed at Site 4 (mean TCC 54,372 cells/mm^2^ and mean ICC of 33,841 cells/mm^2^ during Week 12 sampling), which is a surface water site located 27 km into the network. The site that observed the lowest peak cell count was Site 2, reaching a maximum TCC of 3,273 cells/mm^2^, and mean ICC of 1,605 cells/mm^2^ by Week 12. Site 2 is supplied by ground water and is only 6 km into the network.

Biofilm TCC and ICC at Site 2 exhibited less variation than cell counts obtained from Site 3, however, similar temporal trends were observed. Both TCC and ICC within the biofilm at Site 2 increased from Day 0 to Week 4 reaching a mean TCC of 1,049 cells/mm^2^ and mean ICC of 671 cells/mm^2^. Similar to Site 3, biofilm cell counts at Site 2 declined between Weeks 4 and 6, before increasing again between Weeks 8 and 12. Biofilm cell counts at Site 2 reached a maximum TCC of 3,273 cells/mm2, and mean ICC of 1,605 cells/mm^2^ during Week 12.

TCC of biofilms formed in the BMD installed at Site 3 exhibited an interesting pattern, with a clear increase in TCC from Day 0 to a peak of 11,304 cells/mm^2^ at Week 4. Following this, there was a sharp decrease in TCC to a mean of 574 cells/mm^2^. The TCC became more stable following Week 6, with a steady increase in TCC up to a peak at Week 12. ICC were found to be more consistent during the 12 week fouling period, reaching a peak average cell count of 1,507 cell/mm^2^ at Week 10.

Similarly, the TCC at the final site, Site 4, increased from Day 0 to a peak of 9,551 cells/mm^2^ at Week 3, before declining to a mean TCC of 2,746 cells/mm^2^ at Week 4. The TCC then increased from Week 6 until reaching a mean TCC of 54,372 cells/mm^2^ at Week 12. ICC exhibited a roughly exponential trend in ICC, increasing from Day 0 to a mean ICC of 33,841 cells/mm^2^ at Week 12.

#### Bulk Water Quality at Sites 2, 3, and 4

To investigate trends observed during the biofilm cell count sampling at Sites 2, 3, and 4, the bulk water quality at each BMD outlet was collected and analysed over the same 12-week time period. Bulk water quality values supplying the BMD installed at Site 2, Site 3, and Site 4 are presented in Table 3. The bulk water TCC and ICC was found to be lowest at Site 3, potentially a result of the highest free and total chlorine concentration observed here (total chorine median = 0.74 mg/L; free chlorine median 0.42 mg/L). The TCC within bulk waters was similar at Site 2 and Site 4, but the ICC was significantly highest at Site 4. This does not correlate with the rank order of the BMD quantification of fouling rates, supporting the prior assumption and previous evidence that such data is insufficient to indicate biological fouling rates.

**FIGURE 5:**
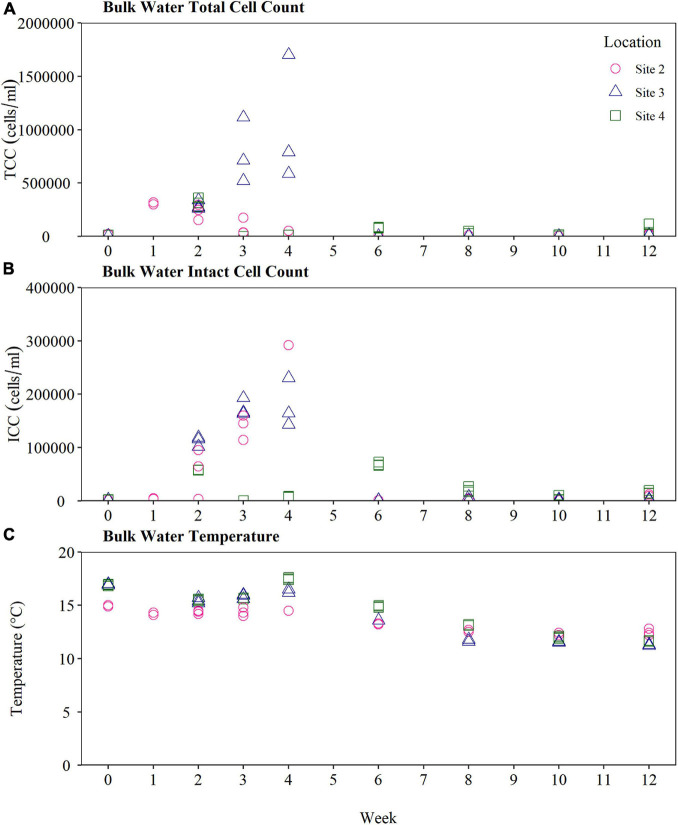
Bulk water quality supplying the biofilm monitoring device at Site 2, Site 3, and Site 4. **(A)** Total cell count and **(B)** intact cell count **(C)** water temperature of the bulk water. Bulk water was samples weekly for the first 4 weeks, and every 2 weeks from Weeks 4 to 12 (n-3 in all cases). TCC, total cell count; ICC, intact cell count.

Raw TCC and ICC time series results from the bulk water at Site 2, Site 3, and Site 4 is presented in Figure 5. The TCC within the bulk water supplying the BMD at Site 3 experienced a large spike at Weeks 3 and 4 (Figure 5), before decreasing again by Week 6. The same trend was also observed in the biofilm cell count data at Site 3, highlighting interactions between planktonic and biofilm microbiology.

The water temperature within each of the BMDs at Site 2, Site 3, and Site 4 was found to be relatively stable throughout the 12-week sampling period. The water temperature at Site 2 exhibited the smallest change in temperature, with a range of 12.2–15.0°C, compared to a range of 11.2–17.0°C at Site 3 and a range of 11.6–17.6°C at Site 4. In all cases the maximum water temperature at each site was recorded at Week 4, reaching a peak temperature of 17.6°C at Site 4, 17.0°C at Site 3 and 15°C at Site 2. Air temperatures also exhibited a seasonal high around weeks 3 and 4 at all sites, reaching value of 24.0°C at Site 2, 31.0°C at Site 3 and 35.5°C at Site 4, compared to 17.4°C at Site 2, 18.0°C at Site 3, and 17.1°C at Site 4 typically in the weeks before and after. All sites exhibited a gradual decrease in temperature from Weeks 4 to 12, consistent with United Kingdom seasonal transition from Summer to Autumn.

#### Historical Water Quality and Customer Contact Data

**FIGURE 6:**
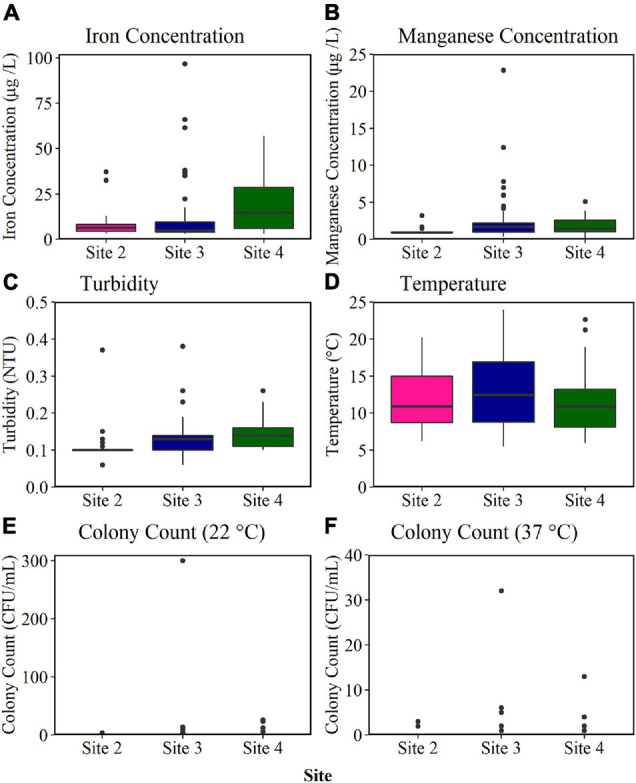
Historical bulk water quality data obtained from random daytime sampling of district metered areas (DMAS) supplied by Site 2, Site 3, and Site 4 trunk mains for the last 6 years. Median, interquartile range and range are plotted. Outlier values are marked with a black circle. NTU, nephelometric turbidity unit; CFU, colony-forming unit; Iron *n* = 27 Site 2; *n* = 66 Site 3; *n* = 32 Site 4. Manganese *n* = 26 Site 2; *n* = 66 Site 3; *n* = 32 Site 4. Turbidity *n* = 25 Site 2; *n* = 63 Site 3; *n* = 27 Site 4. Temperature *n* = 61 Site 2; *n* = 144 Site 3; *n* = 49 Site 4. Colony count (22°C) *n* = 27 Site 2; *n* = 68 Site 3; *n* = 29 Site 4. Colony count (37°C) *n* = 27 Site 2; *n* = 43 Site 3; *n* = 28 Site 4.

The biofilm cell count data obtained from the BMD installed at Site 2, Site 3, and Site 4 (Figure 4) indicated different biofouling rates. The association of biofilm with discolouration events (section “Introduction”) would hence suggest an increasing discolouration potential across the sites in the order 2, 3, and 4. The BMD results were therefore compared to historical water quality and customer contact data to determine if this trend was supported. The median and range of bulk water parameters sampled from DMAs supplied by the monitored trunk mains (Site 2, Site 3, and Site 4) during regulatory random daytime sampling over the last 6 years are presented in Figure 6. Of the parameters sampled, only turbidity, iron and manganese were found to be statistically significant across the three sites (*p* < 0.05). The median turbidity was highest at Site 4 (0.14 NTU), mid-range at Site 3 (0.13 NTU), and lowest at Site 2 (0.10 NTU). The median iron concentration was also found to be highest in bulk water at Site 4 (14.65 μg/L), but mid-range at Site 2 (5.00 μg/L), and lowest at Site 3 (6.50 μg/L). In contrast, the median manganese concentration was highest at Site 3 (1.70 μg/L), and only 1.45 μg/L at Site 4, and 0.90 μg/L at Site 2. The median water temperature and colony counts were similar across all three sites, exhibiting no statistical difference. As a result of this analysis no consistent site-specific trends could be drawn from the historical bulk water quality data with respect to water quality performance. This is perhaps unsurprising as the mobilisation of biofilm, and discolouration, requires a hydraulic event (e.g., increased demand, pipe burst) to generate an excess shear stress. Such events are sporadic and unlikely to be captured by regulatory sampling.

**FIGURE 7:**
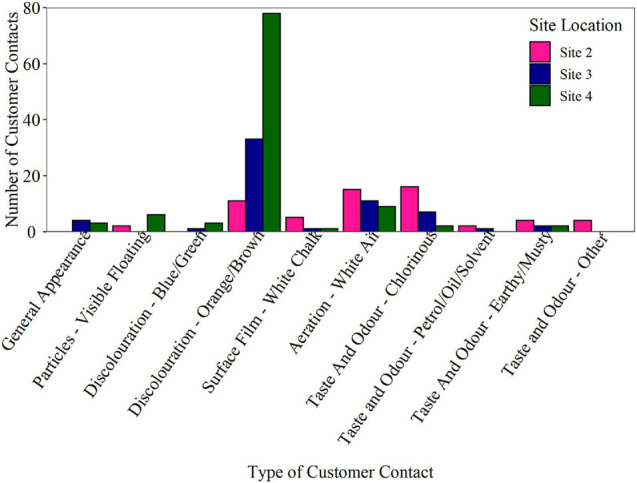
Customer contact numbers for the district metered areas (DMAs) supplied by Site 2, Site 3, and Site 4 trunk mains for the last 6 years. Over this time period, 59 customer contacts were received at Site 2, 90 at Site 3 and 130 customer contacts at Site 4. Raw data is plotted as the population served by each trunk main is similar (4,342 persons at Site 2, 5,021 persons at Site 3, and 6,226 persons at Site 4).

To investigate if the biofouling rates derived from the BMD delivered insight into network performance, historical customer contacts were analysed for each network. Customer contact numbers for DMAs supplied by Site 2, Site 3, and Site 4 trunk mains for the last 6 years are presented in Figure 7. Raw data is plotted as the data was collected from areas serving similar populations. Customer contacts include both appearance and taste and odour, however, the proven association between biofilms and discolouration adds weight to relevance of this category. At Site 4, a total of 78 discolouration (orange/brown) customer contacts were reported during the 6-year period, in comparison to 33 at Site 3 and only 11 at Site 2, i.e., Site 4 had 7 × the number of customer contacts in the last 6 years compared to Site 2. A review of the data indicated that customer contacts were spread over the period with no evidence of significant clustering in any network to suggest a single major event. These results suggest that Site 4, with the highest BMD measured biofouling rate, also had the highest incidence of discolouration events. Site 2 which exhibited the lowest BMD biofilm cell counts exhibited the lowest number of customer contacts due to discolouration. Importantly, the results obtained from the BMD only took 12 weeks of data collection (only 6 weeks for differences, however, to become apparent) compared to the many years required to collate sufficient water quality or customer contact data. It should be noted that the BMD is representative of a single point, while customer contacts will be more of a holistic measure of the wider network area, including being influenced by pipe material, length and condition throughout the DMAs. For example, the DMAs served had different percentages of cast iron pipe: Site 2 43%, Site 3 16%, and Site 4 50%. However, this information does not correlate with or explain either the bulk water iron data or the discolouration customer contact data.

### The Impact of Seasonality on Biofouling: Site 3

**FIGURE 8:**
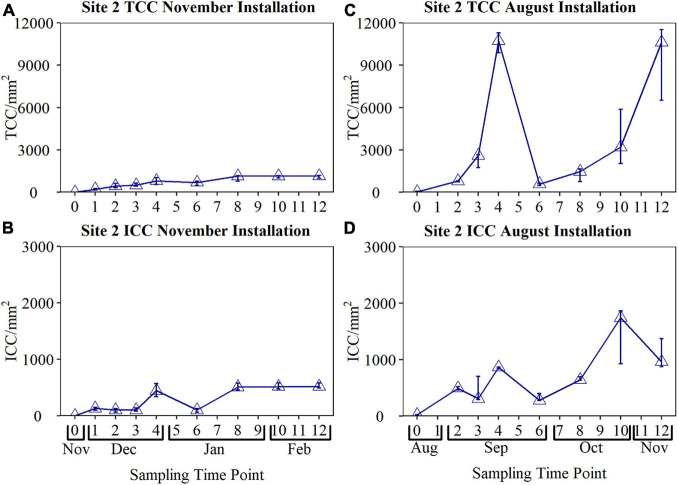
Comparison of biofouling curves obtained from the biofilm monitoring device installed at Site 2 during the November and August installations. Biofilm samples were collected weekly for the first 4 weeks, and every 2 weeks from Weeks 4 to 12 (*n* = 3 in all cases, raw data is plotted). TCC, total cell count; ICC, intact cell count.

To determine if there was any effect of seasonality in biofouling rates obtained from the BMD, two sets of data were collected from the same site (Site 3) at different times of the year. The BMD was first installed in November for 12 weeks, before being reinstalled in August and again sampled for 12 weeks (Figure 8). Bulk water quality data during the November and August installations is provided in Table 3 and Supplementary Table 1. The median temperature during the November installation was 8.8°C (max 11.3°C, min 7.6°C), and 14.4°C (max 17.0°C, min 11.2°C) during the August installation. The biofilm cell counts observed in the BMD during November and August were found to be statistically significant, both for TCC (*p* = < 0.001) and ICC (*p* = < 0.001). The cell counts obtained from the BMD reached a peak TCC of 11,535 cells/mm^2^ and peak ICC of 1,858 cells/mm^2^ during the August installation, in contrast to a peak TCC of only 1,180 cells/mm^2^, and peak ICC of 582 cells/mm^2^ during the November installation.

Examining the November fouling rate, TCC increased from Day 0 to 936 cells/mm^2^ by week 4. ICC increased only slightly between Day 0 and week 3, before a large increase up to 494 cells/mm^2^ by week 4. During the November installation, both the TCC and ICC decreased significantly at week 6. Surprisingly, this same trend, characterised by an increase in cell counts up to Week 4, before a dramatic decrease in cell counts at Week 6, was observed during both installations. During the November installation, both TCC and ICC reached a plateau at Week 8, remaining relatively constant until Week 12.

## Discussion

Installation of the BMD at three operational DWDS found that different biofouling curves were generated when the BMD was supplied by different waters (Figure 4). This indicates that the BMD can be used to assess biofouling rates using biofilm cell concentrations. The conditions within each BMD (flow rate, material, and configuration) were consistent between sites, to enable a direct comparison of the biofouling rates in the different DWDS. A positive correlation was observed between BMD biofouling rate, as assessed using biofilm TCC and ICC, and historic discolouration customer contacts. Thus suggesting a link between biofilm cell counts, the biofouling rate and potential discolouration response. Pick et al. (2021) also observed a positive relationship between biofilm cell counts, and discolouration risk within an experimental pipe loop facility. However, the discolouration risk posed by a biofilm is not only a function of biofilm cell counts, but also EPS quantity and structure, and the community composition of the biofilm (Fish et al., 2016). The EPS provides structure and mechanical stability through a variety of processes including hydrophobic, electrostatic and dispersive interactions (Flemming et al., 2007; Neu and Lawrence, 2009), hence it is a critical component of the biofilm. The amount of EPS produced by a biofilm is dependent on various factors such as the species of organisms (microbiome composition), biofilm age (Kokare et al., 2009) and hydraulic regime (Fish et al., 2017). A study by Fish et al. (2020) found that TCC and ICC mobilised into the bulk water from biofilms was inversely correlated with turbidity and metals (iron and manganese) concentrations during flushing trials within an experimental DWDS. Similarly, Prest et al. (2021) found hydraulics impacted particle counts and turbidity, but not TCC and ICC in the bulk water within operational DWDS. Although the EPS and community composition within the biofilm is likely to influence the biofouling rate, current analysis approaches are time consuming, costly and require substantial biomass for accurate data. Monitoring the change in biofilm cell abundance over time using flow cytometric analysis of biofilm samples from the BMD is rapid and still informative, likely indicative of an increase (or change) in other biofilm components such as the EPS, although this is a crucial area that needs further research.

TCC and ICC results obtained from the BMD exhibited a similar trend within sites, although at different magnitudes of absolute cells, with biofilm cell counts at Site 2 and Site 3 increasing between Day 0 and Week 4, declining between Weeks 4 and 6, before increasing again between Weeks 8 and 12. A similar trend over time in planktonic TCC and ICC at Site 2 and Site 3 was observed (Figure 5), with an increase in planktonic TCC and ICC up until Week 4, before a dramatic decrease in cell counts. This same pattern of biofilm growth, followed by loss of cells, and then cell recovery was also exhibited in the TCC at the final site, Site 4. As this trend was observed at all sites, this potentially suggests a shift in the biofilm community composition from initial colonisers, to a more stable community composition. Douterelo et al. (2018) observed a similar trend in drinking water biofilms growth, reporting that a 1-month biofilm development stage was followed by a temporary decrease in biofilm diversity and cell volume, before the biofilm recovered. Further research is required to understand the driving interactions leading to this observed shift in biofilm cell counts. In most of the results presented here the ICC, which is commonly attributed as the viable population, was found to be the more stable and consistent measure of biofilm and bulk water cell counts than TCC. The relationship between TCC, ICC and biofilm growth and hence biofouling is complex and underexplored. Whilst there are practical benefits to monitoring only one of the classifications (TCC or ICC) in the field, substantially more data and research is needed to inform which parameter is most meaningful and useful for indicating biofouling within DWDS.

This study found that when comparing the biofouling rate obtained from the same DWDS location, but at a different time of year, the biofouling rate was greater when the water temperature was higher (Figure 8). Temperature is known to be an important determinant of drinking water quality as it affects the physical, chemical and biological processes occurring within the DWDS, including microbial growth and the rate of chlorine decay (Agudelo-Vera et al., 2020). This research showed that the growth rate of attached microorganisms increased during the warmer summer months. If biofilm behaviour is analogous to that of discolouration layers within the DWDS, this suggests that the discolouration risk posed by biofilm would be higher in the warmer months. In a 1-year study investigating discolouration risk within operational trunk mains, Sunny et al. (2018) observed a positive relationship between temperature, material accumulation and total organic carbon (TOC). This finding indicated that organic material, and therefore likely associated microbial behaviour, played a critical role in the discolouration risk posed by a DWDS. Further supporting this finding, Cook et al. (2015) found that a greater numbers of discolouration complaints were made during higher temperatures in the United Kingdom, with fewer in the winter when water temperatures typically fall to <6°C. Although this and previous studies focus on temperature as a dominant seasonal effect, and temperature is likely to be accelerating the biological process occurring within DWDS, there will also be seasonal changes in the source and treated water qualities entering the systems, such as TOC as noted by Sunny et al. (2018). Similarly, Pick et al. (2021) noted seasonal changes in assumable organic carbon and DWDS performance.

The BMD with the highest water age (16 h), and supplied by the highest concentration of TCC and ICC in the bulk water (Site 4), reached the highest peak of TCC and ICC within the biofilm. This supports the observation that water quality is a function of hydraulic retention time (HRT) or water age, with water deteriorating as it travels away from the WTW due to complex interactions occurring within the DWDS (Machell and Boxall, 2014). It should be acknowledged that the results from the BMD are indicative of biofouling based on bulk water characteristics at the location of installation, and not due to differences in hydraulics as all the BMD were operated under the same steady state flow (1 L/s). Further research is need to establish if the biofilms which accumulate within the BMD are fully representative of the biofilm community composition and physical structure found within the DWDS, which will be impacted by hydraulic parameters. The data presented here also showed sensitivity to a short “heat wave,” and different levels of impacts of these across the sites. This indicates that caution is needed to check for such events, particularly if the overall monitoring period were to be reduced.

A key finding of this research was that the biofouling rates obtained from the BMD seemed to correlate with historic discolouration customer contacts. This suggests that the BMD was able to identify the site that potentially posed the greatest discolouration risk after 12 weeks of sampling, compared with the many years required to collect indicative customer contact data. However, a greater range of sample sites, with contrasting water qualities and historic customer contact data, would be needed to substantiate this finding further. This difference could have been reliably assessed at less than 12 weeks from the data presented here, 6 weeks of data was needed for visible site differences, but further application to a wider range of sites would be necessary to fully confirm the minimum necessary period. A number of studies have tried to quantify the discolouration potential within different DWDS using the rate at which material accumulates on pipe walls following repeated flushing of pipes and detailed monitoring at specified time intervals (Husband and Boxall, 2010; Blokker and Schaap, 2015). Such approaches require detailed planning, significant water discharge and largely focus on DWDS physical components, yet these are complex environments in which physical, chemical and biological parameters associated with discolouration, continuously interact (Husband et al., 2016). The established links between biofilms, discolouration and water quality highlight how a better understanding of biofouling rates and changes in these growth rates following capital or operational changes, could help inform water quality management strategies.

The BMD developed in this study has the practical benefit of being able to provide water utilities with a quantitative risk ranking of locations within network in 12 (or less) weeks that provides good correlation with historic customer contacts. This is significant as such customer contact data must be collected over many years, and is hence a very lagged indicator. However, it should be noted that the device is indicative of a single location and conditions at that point, whereas customer contacts will be representative of a wider area including the impacts of pipe materials and conditions. Furthermore, this research, and potential uptake of the device by water utilities, could be used to assess the impact of operational changes including maintenance scheduling and to review network performance post capital or operational network changes. Such information, and insight into the connectivity of WTW and DWDS, has not previously been possible, but is vital for the long term holistic management of water supply systems, and informing the trade-off between capital (treatment works improvements) and operational (network flushing) expenditure.

A comparison of biofilm data obtained from the BMD to historical water quality data obtained from the same operational DWDS found no clear correlations between parameters (Figure 6). This highlights that although historic water quality provides insight into the pipeline environment, it does not represent the biofouling rate at the pipe wall as this is governed by more complex processes. The DWDS with the highest BMD growth (and hence biofouling) rate (Site 4) also had historically the highest iron concentration and turbidity values obtained from random daily sampling over the last 6 years. However, the site with historically the highest manganese concentration within the bulk water was Site 3, which had the second highest biofilm forming rate. Iron, manganese and turbidity are all parameters associated with discolouration events (Seth et al., 2004). Although it has been suggested that microbial biofilms may play a critical role in discolouration (Ginige et al., 2011; Fish et al., 2020), the relationship between biofilms and the accumulation of inorganic particles, such as iron and manganese, in DWDS is not fully understood. The results presented here show a strong correlation between biofilm cell count data and number of discolouration customer contacts, yet inconsistent trends with historical water quality data. This finding highlights that sampling both the bulk and biofilm within DWDS environments provides a far greater value than sampling the bulk water alone, and is essential to gain a holistic understanding of the discolouration risk posed by a DWDS.

## Conclusion

This research has developed a non-destructive, non-disruptive, and easily deployed monitoring tool and procedures to measure biofouling rates from within typically inaccessible Drinking Water Distribution Systems (DWDSs). The BMD was first verified in the laboratory to confirm that the optimum length, orientation and flow rate of the device to produce consistent results, the BMD was then successfully validated by installing within operational DWDS. The BMD assessed different biofouling rates at the sites studied and these rates were found to have a positive correlation with the number of discolouration customer contacts, suggesting this is a viable and rapid technique to assess network performance. No correlation was found between the rate of DWDS biofouling and historic bulk water quality data, providing evidence of the understanding that sampling the bulk water alone cannot be used to reliably inform what is happening at the pipe wall. The results obtained from the BMD took 12 weeks of data collection (6 weeks of data was needed for site differences to become apparent) compared to a number of years required to collect and collate customer contact data. The BMD has the potential to rank network performance, investigate seasonal effects and evidence impact of operational changes or capital investment. With improved understanding and tracking of the fouling rate within DWDS, networks could be prioritised in terms of risk factors and return rates for pro-active maintenance or future investment identified to safeguard water quality.

## Data Availability Statement

The original contributions presented in the study are included in the article/Supplementary Material, further inquiries can be directed to the corresponding author/s.

## Author Contributions

FP, KF, SH, and JB conceived and designed the experiments, analysed the data, and wrote the manuscript. FP and KF performed the experiments. All authors contributed to the article and approved the submitted version.

## Conflict of Interest

The authors declare that the research was conducted in the absence of any commercial or financial relationships that could be construed as a potential conflict of interest.

## Publisher’s Note

All claims expressed in this article are solely those of the authors and do not necessarily represent those of their affiliated organizations, or those of the publisher, the editors and the reviewers. Any product that may be evaluated in this article, or claim that may be made by its manufacturer, is not guaranteed or endorsed by the publisher.

## Supplementary Material

Supplementary Figure 1 Photograph of the biofilm monitoring device (BMD) installed at an operational field site, including tube sections, connectors and a flow valve on the BMD outlet.
