# Supplementary figures and images for: Non-invasive Biofouling Monitoring to Assess Drinking Water Distribution System Performance
Source: Front Microbiol. 2021 Oct 28;12:730344. doi: 10.3389/fmicb.2021.730344 (PMC8581547; doi:10.3389/fmicb.2021.730344)

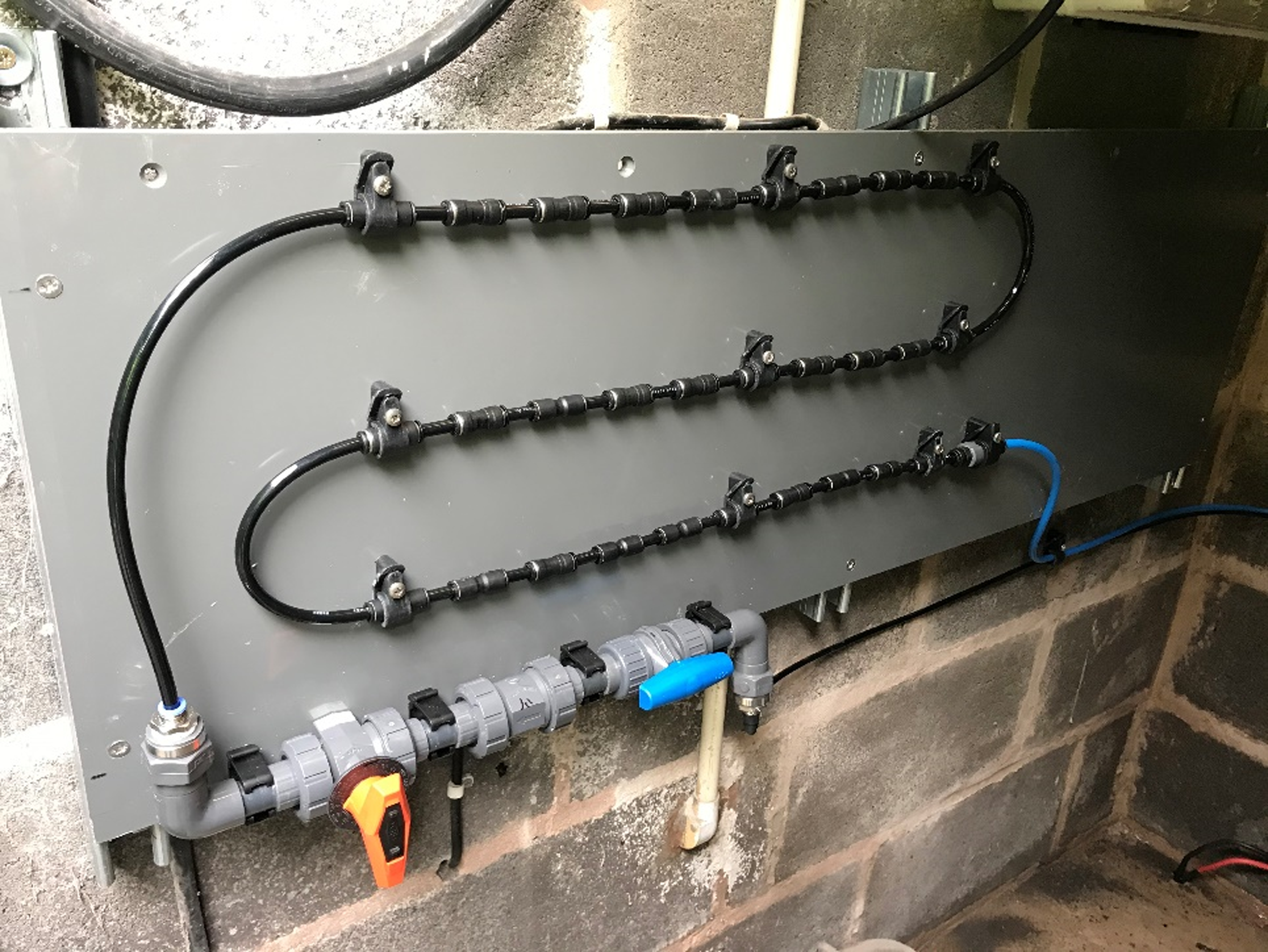

Supplement: Supplementary Figure 1 — Photograph of the biofilm monitoring device (BMD) installed at an operational field site, including tube sections, connectors and a flow valve on the BMD outlet. [file Image_1.TIFF]
