# Supplementary material for: Non-invasive Biofouling Monitoring to Assess Drinking Water Distribution System Performance
Source: Front Microbiol. 2021 Oct 28;12:730344. doi: 10.3389/fmicb.2021.730344 (PMC8581547; doi:10.3389/fmicb.2021.730344)
Supplement: Supplementary file 2 [file Table_1.DOCX]

**Supplementary Table 1: Bulk water quality supplying the biofilm monitoring devices at Site 3 from the November installation.** Median and range of bulk water parameters collected in triplicate are listed. TCC: total cell count; ICC: intact cell count. Site 3 water quality was sampled for 12 weeks from November – February. *Turbidity not sampled at Site 3 during the November installation due to equipment failure.

|  | **Site 1** | |
| --- | --- | --- |
| **Parameter** | **Median** | **Range** |
| TCC (cells/mL) | 1124 | 872-1229 |
| ICC (cells/mL) | 320 | 270-392 |
| Total Chlorine (mg/l) | 0.82 | 0.68-0.98 |
| Free Chlorine (mg/l) | 0.51 | 0.40-0.62 |
| Temperature (°C) | 9.1 | 7.6-11.3 |
| pH | 6.53 | 6.35-6.78 |
| ORP (millivolts) | 506 | 495-522 |
| Turbidity (NTU) | * | * |
